# Diagnostic Challenges and Influencing Factors in Non‐Melanoma Skin Cancers: A Retrospective Analysis of Basal Cell Carcinoma and Squamous Cell Carcinoma Cases

**DOI:** 10.1002/cnr2.70332

**Published:** 2025-09-04

**Authors:** Nicholas Florin Kormos, Ioana Daria Paval, Carina Maria Petrenciu, Alin Stefan Vizitiu, Adrian Lucian Baican

**Affiliations:** ^1^ Dermatology Department, Faculty of Medicine “Iuliu Hatieganu” University of Medicine and Pharmacy Cluj‐Napoca Romania

**Keywords:** basal cell carcinoma, basosquamous carcinoma, non‐melanoma skin cancer, SARS‐CoV‐2, squamous cell carcinoma

## Abstract

**Background:**

Non‐melanoma skin cancers (NMSC) are the most frequent cutaneous tumors globally. Basal cell carcinoma (BCC) and squamous cell carcinoma (SCC) represent the most frequently encountered representatives of this group and may represent a diagnosis challenge in some circumstances of hard to differentiate tumors.

**Aims:**

The aim of this study was to determine the factors that influence the diagnosis of NMSC and their impact.

**Methods and Results:**

A single center, descriptive, retrospective study was performed, with a total of 866 tumors excised from 678 patients between 2016 and 2022. Cases were then analyzed based on their histological diagnosis and characteristics and the available clinical data. From the 866 cases, 709 were histologically diagnosed as BCC and 157 as SCC. The clinical accuracy for BCCs was 95% whereas for SCCs was 62% with both having large variations among attending dermatologists. Clinical factors that were found to influence the diagnosis were: advanced age (*p* ⟨ 0.001), a biopsy beforehand (*p* = 0.009), and multiple diagnosis or uncertainty (*p* = 0.005) were predictive for a final diagnosis of SCC. Factors predicting BCCs were: the presence of multiple lesions (*p* = 0.032), presence of ulcerations (*p* ⟨ 0.001), age under 50 (*p* = 0.002). BCCs were more likely to be correctly diagnosed than SCC (*p* ⟨ 0.001). Cases with multiple BCCs were thinner compared to single‐lesion excisions (*p* ⟨ 0.001). A tumor reduction was observed after the 2020 the SARS‐CoV‐2 (*p* = 0.023).

**Conclusions:**

Differentiating NMSC can be challenging, but several trends have been observed which may aid in further improving the diagnosis rates especially for SCCs.

## 1 Introduction

Basal cell carcinoma (BCC) and cutaneous squamous cell carcinoma (SCC) are two of the most common skin cancers and non‐melanoma skin cancers (NMSC) [1, 2]. Both are keratinocyte carcinomas, as they both originate from the epidermal keratinocytes [2]. Besides the genetics and epigenetic regulation, carcinogenesis can be influenced by a variety of risk factors, out of which advanced age, prolonged and cumulative exposure to ultraviolet rays (especially UVB), ionizing radiation, immunosuppression, a Fitzpatrick skin type of I and II, and carcinogenic substances (like arsenic) were the most reported ones [3, 4, 5].

Although BCC has low mortality and an insignificant chance of metastasis by blood or lymphatics, the morbidity is high, due to the ability of local tissue infiltration and destruction [1, 2, 6]. In contrast with BCC, SCC has a greater propensity to metastasize and is responsible for the majority of NMSC‐related deaths [4, 5, 7, 8, 9].

Because the clinical profiles of BCC and SCC are widely heterogeneous in relation to the anatomical site, dimensions, definition of the margins, pigmentation, and tumor grade differentiation, NMSCs can easily mimic other benign or malignant lesions, even when they do not pose atypical features [8, 10]. Therefore, an accurate diagnosis can be challenging for dermatologists, and errors might occur [11].

Clinically, BCC and SCC do not have a universal classification. BCCs can be subdivided into: nodular, superficial, fibroepithelial, and morpheaform [4, 6]. The clinical presentation of BCC mostly includes a translucent and smooth pearly structure (papule or nodule), with telangiectatic vessels and spontaneous bleeding, a high inclination towards ulceration, central depression, and different coloration (pink, white or skin‐colored lesions), but varieties are also reported in the form of macula or atrophic plaque appearance and scar‐like lesions [2, 3, 6, 7].

Common SCC types include the superficial, nodular, plaque, and flat forms [3, 6]. SCC is typically seen as a solitary scaly or crusty reddish hyperkeratotic papule or plaque, with ulceration, a smooth surface, and a firm aspect [2, 10]. Clinical features may include bleeding, pruritus, pain, paresthesia, or asymptomatic lesions, depending on the perineural invasion, tumor size, depth of invasion, and differentiation [9, 11].

Histopathologically, BCC and SCC have numerous subtypes. BCCs are described as a relatively circumscribed tumoral growth arising from the epidermis, and depending on the form, with basaloid nests [2, 3, 6, 7]. SCCs have been described as low to high‐risk histological types, with common features including nests of atypical squamous epithelial cells penetrating into the dermis [6, 10].

Noninvasive imaging tools including dermoscopy, reflectance confocal microscopy, ultrasonography, optical coherence tomography, and cross‐polarized light and fluorescence photography can provide better accuracy, reducing the diagnostic uncertainty [2, 9, 11]. Dermoscopy is the most accessible form of clinical examination with hallmarks of BCC including: vascular structure (arborizing vessels), network areas of melanocytic lesions, ovoidal shape of blue‐gray colored nests, and ulceration [5, 9, 11]. Typical SCC features include red or unpigmented color, erosions or ulceration, keratotic plugs or central keratin mass, and diverse vascular structures with dotted, glomerular, linear irregular, and hairpin vessels or red starburst pattern [7, 8, 11].

Most BCC and SCC cases occur on photoexposed areas such as the head and neck; early detection can significantly influence the cosmetic results, which together with complete neoplasm excision and preserved functional capacity reflect the main goals of NMSC treatment [2, 10].

Surgery is the most effective treatment for BCC and SCC. Mohs micrographic surgery is considered the gold standard but is not universally available, and the most common form of surgical treatment remains the wide local excision. Although several other therapeutic options are also available for ineligible patients to first‐line treatment options such as curettage and cautery, radiotherapy, immunotherapy, cryosurgery, photodynamic therapy, topical treatments, and systemic therapy [2, 4, 12]. By mismatched or delayed diagnoses, BCCs and SCCs can be wrongfully treated, affecting the likelihood of healing and leading to a necessity of more complex or avoidable surgical interventions, thus increasing the psychological, financial, and cosmetic burden of NMSCs [9, 13].

Despite the recent advances both in new diagnostic tools and the improvement of already consolidated tools, the clinical challenge for a correct differential diagnosis for some skin cancers remains constant [7, 14]. A clinical misdiagnosis might aggravate the patient's prognosis or lead to an increased chance of complications [10, 12]. While the clinical and dermoscopical features for BCCs and SCCs are widely recognized by trained dermatologists, time constraints and other factors might impede a correct diagnosis [2, 11]. Therefore, a need for a better understanding of clinical features that might indicate the correct clinical diagnosis and optimize the use of resources while reducing potential complications is needed.

The aim of our study was to determine both the clinical and histopathological accuracy of BCC and SCC cases of a large, multi‐year cohort, as well as present the clinical and demographic factors that influenced the diagnostic rationale, and consequently, the therapeutic approach.

## 2 Materials and Methods

**FIGURE 1:**
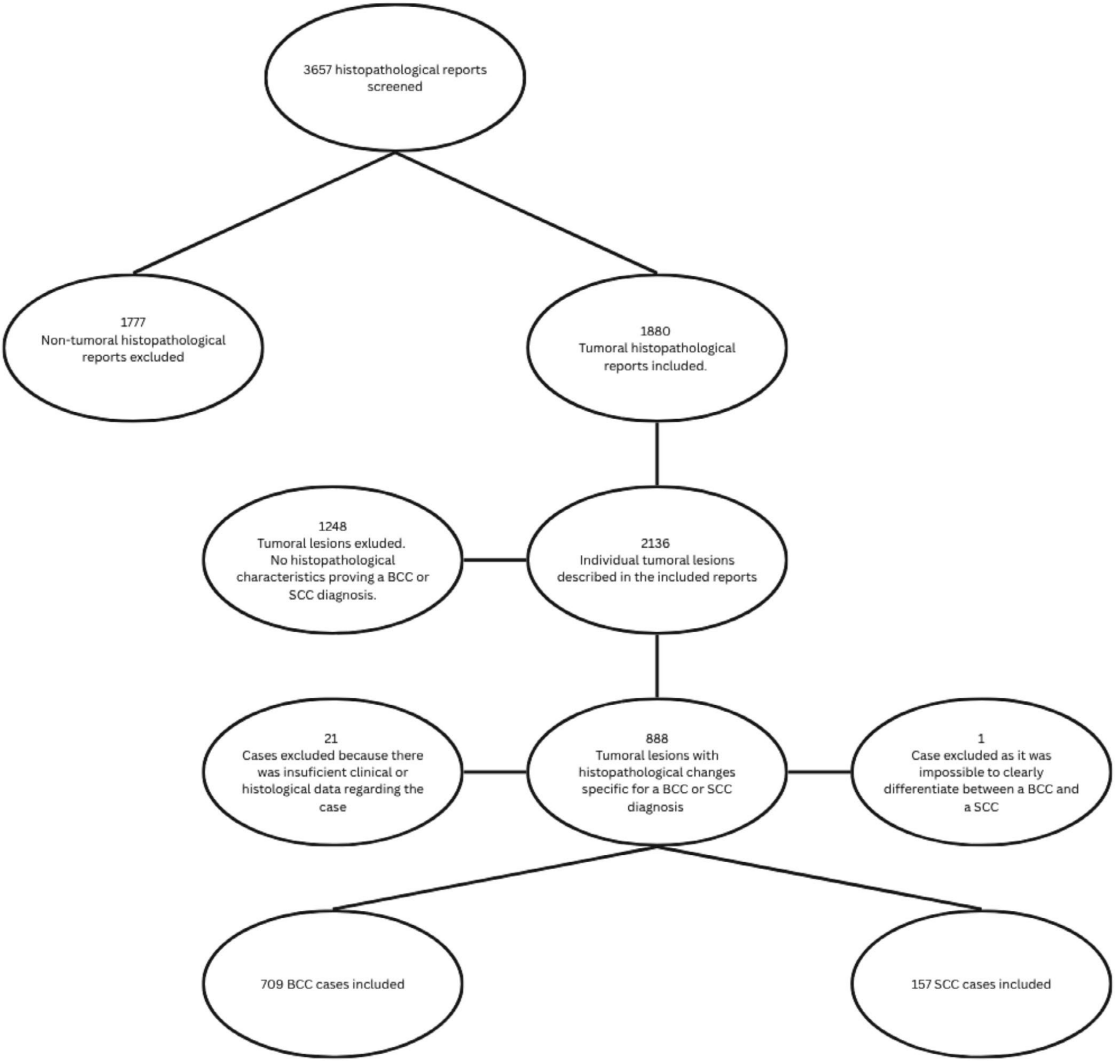
Patient selection flow‐chart detailing the inclusion of cases into the study. BCC, basal cell carcinoma; SCC, squamous cell carcinoma.

A single center, retrospective, descriptive study was performed. The cases were selected from 3657 histopathological reports of lesions excised during 2016 and until 2022. All included cases had a complete histological and clinical report; biopsies and complete excisions were included, with cases having a biopsy and a follow‐up excision being considered as a singular lesion and counted once. Cases of immunosuppressed patients or those that had several previously excised tumors were not excluded, but their total number was negligible. We excluded cases with incomplete or lacking clinical data or a histopathology report (Figure 1). All included cases were extracted from the Cluj Emergency County Hospital database. Written informed consent was obtained from all participants before their inclusion in the study. The study was approved by the Cluj Emergency County Hospital Ethical Board (Approval No 45182/26.11.2024).

Cases were divided into two groups based on their histological diagnosis. The first group consisted of 709 tumors diagnosed as BCC and the second group consisted of 157 SCC. A total of 866 tumors were excised from 678 patients, with some patients having more than one tumor excised. The histological diagnosis was conclusive in all except one case, which was excluded. Immunohistochemistry was performed for 39 cases in total, out of which 10 cases were SCCs and 29 BCCs.

Each case was evaluated at least once in a preoperative setting and at least once when the referral for surgery or biopsy was made. The clinical evaluation included dermatoscopical evaluation in all cases performed both by the attending physician and a dermatologist in training. The excisions and biopsies were performed as a day procedure or through hospitalization in an operating room. Histopathological examination was performed in the same hospital with a clinical description of the lesion but blinded to the clinical image of the lesion.

Some of the cases evaluated in our study could not be clinically diagnosed, and the surgical protocol was established as the most probable diagnosis to have a working diagnosis for the surgical excision, margins, and further recommendations until the definitive diagnosis was established. Therefore, the clinical diagnosis discussed in the study may not have been the actual diagnosis the patient received before and shortly after the surgery. Cases where the clinical diagnosis was not certainly defined or where there was ambiguity regarding the diagnosis with one or several other differential diagnoses included were defined as having an uncertain diagnosis.

The primary endpoint was diagnostic concordance between clinical and histopathological diagnoses of BCC and SCC. Secondary endpoints included associations between diagnostic accuracy and patient demographics, lesion location, tumor thickness, histopathologic features, and physician performance.

To assess possible temporal trends, we divided the study period into a baseline consisting of the years 2018–2019, followed by a one‐year interval for the 2020–2022 period to consider the potential impact of the SARS‐CoV‐2 pandemic on the number of cases and tumor characteristics. Physician performance was analyzed by comparing diagnostic accuracy between the dermatologists present at our clinic during the research interval. All cases were anonymized, and the attending physician's name was available in the records.

### 2.1 Statistical Analysis

The following data was taken into consideration and analyzed: patients' personal identification data, demographics, dermoscopic and clinical characteristics including the size, ulceration, location, number of excised lesions, certain and differential clinical diagnoses, the certain and differential histopathological diagnoses, tumoral dimension, and microscopical characteristics such as immune cell infiltrate, tumoral differentiation, squamous differentiation of BCCs. A temporal trend analysis was performed to evaluate changes in patient numbers and tumor thickness during and after the 2020 SARS‐CoV‐2 pandemic. A value of *p* < 0.05 was considered statistically significant. The following statistical methods were used: Yates corrected Chi‐square test (χ^2^), Odds Ratio (OR), Fischer's Exact Test, and the data was described through the use of mean value (M), standard deviation (SD), standard error of the difference (SEM), 95% Confidence Interval (95% CI), number of cases (N). All the statistical analyses were performed using the IBM SPSS Statistics 29.0.2.0 software environment.

## 3 Results

The mean age of our cohort was 69.88 years with a mean age of 68.62 years (SD = 12.29, SEM = 0.46, *N* = 709) for patients with BCC's and 75.59 years (SD = 10.23, SEM = 0.82, *N* = 157) for patients with SCC. There was a statistically significant age difference between the two, with SCC cases having a more advanced age, with a mean age difference of 6.96 years; 95% CI [4.89, 9.03] was observed (*p* < 0.001). There were 70/709 (10%) and 3/157 (3%) of BCC and SCC cases in patients aged 50 and under; the difference was statistically significant χ^2^(1, *N* = 862) = 9.17, *p* = 0.002, OR = 5.48 [1.76, 27.5], confirmed with the Fischer exact test (*p* < 0.001).

Biopsies were more often performed in SCCs with 21/157 (13%) versus 48/709 (6.7%) for BCCs, χ^2^(1, *N* = 866) = 6.77, *p* = 0.009, OR = 2.13 [1.17, 3.75], confirmed with the Fischer exact test (*p* = 0.013). While the average biopsied SCC was not more advanced, with an average thickness of 3.6 mm compared to a median of 3.36 mm for all SCCs (*p* = 0.88), the mean thickness for BCCs was 1.18 mm (SD = 0.64, SEM = 0.11, *N* = 30) versus a median value of 2.18 mm (SD = 1.68, SEM = 0.06, *N* = 640) for all excised BCCs, the difference was statistically significant, with a mean thickness difference of 1.00 mm, 95% CI [0.41, 1.61] observed (*p* = 0.001). Of the biopsied tumors, 33/69 (48%) were localized on the head, out of which 8/33 (24%) were SCCs and 25/33 (76%) were BCCs.

**FIGURE 2:**
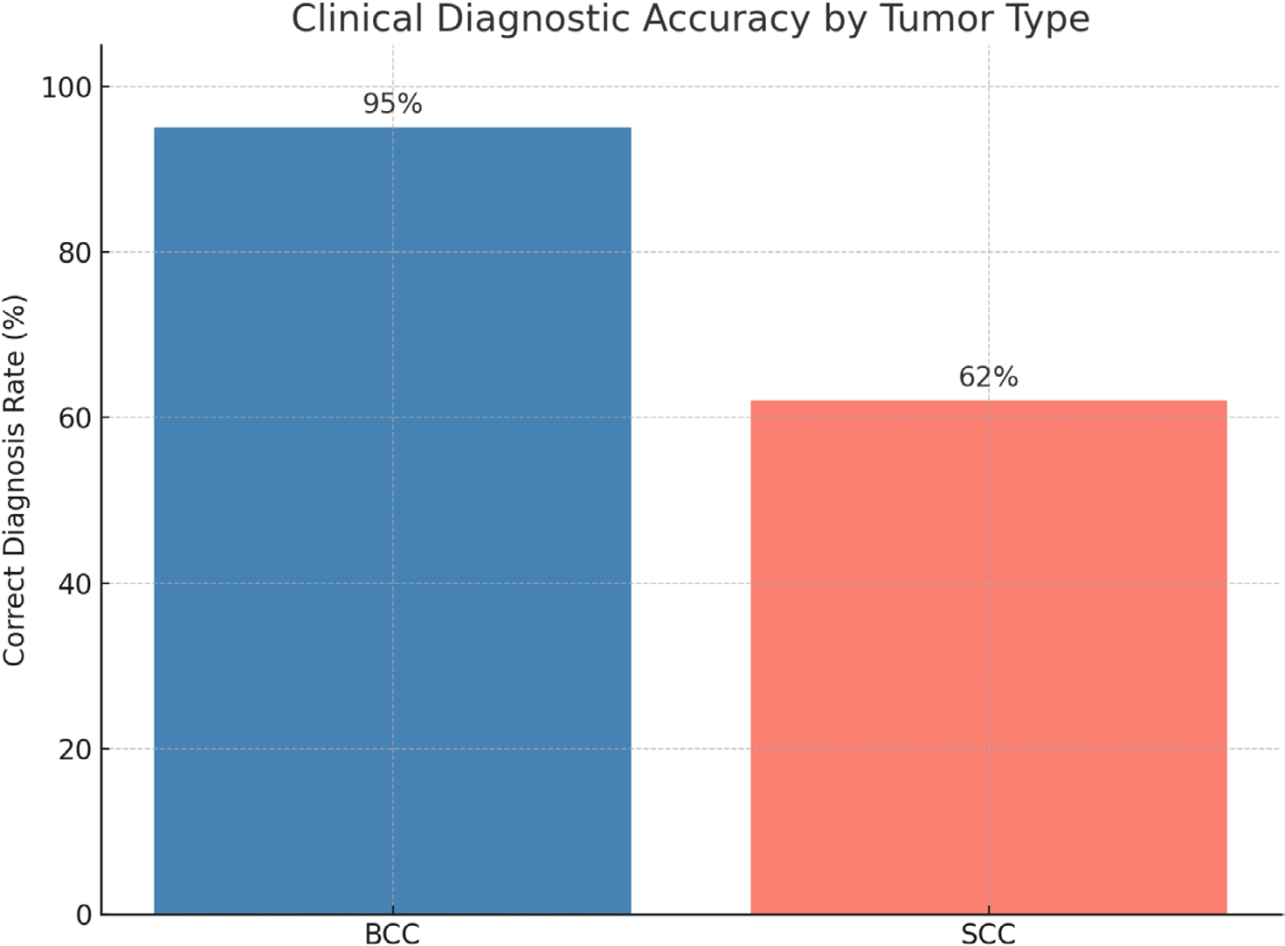
Representation of the clinical diagnosis accuracy by tumor type. BCC, basal cell carcinoma; SCC, squamous cell carcinoma.

In the 709 BCC cohort, there were 675/709 (95%) clinically correct diagnosed tumors, with 655/675 (97%) having a single or certain diagnosis and 20/675 (3%) having a multiple or uncertain diagnosis, and 34/709 (5%) with a mismatch, out of which 31/34 (91%) had a single or certain diagnosis and 3/34 (9%) had a multiple or uncertain diagnosis (Figure 2). The 157 SCCs were 97/157 (62%) clinically correct diagnosed, 88/97 (90%) were certainly diagnosed as SCC, and 9/97 (10%) presented uncertainty. 60/157 (38%) had a clinical‐histopathological mismatch, with 57/60 (95%) having a certain diagnosis, and 3/60 (5%) having an uncertain diagnosis (Table 1). Comparing the correct diagnosis and false diagnosis among BCCs and SCCs, there was a statistically significant difference between the rates of correct diagnosis of the two χ^2^(1, *N* = 866) = 144.9, *p* < 0.001, OR = 12.28 [7.66, 19.68], confirmed with the Fischer exact test (*p* < 0.001).

**TABLE 1:** Description of all clinical and histological diagnoses included in our cohort.

	Clinical			Histopathological	
	Certain diagnosis	Uncertain diagnosis	Misdiagnosis	Certain diagnosis	Uncertain diagnosis
BCC	655	17 (SCC)	22 (SCC)	709	0
		1 (CM)	4 (Nevi)		
		1 (Cyst)	3 (Melanoma)		
		1 (AK)	1 (DF)		
			1 (SK)		
			AK – 1 (AK)		
			1 (Hemangioma)		
			1 (CM)		
Total	655	20	34	709	0
SCC	88	9 (BCC)	32 (BCC)	157	1^a^ (Bowenoid actinic keratosis)
			11 (unspecified)		
			4 (Keratin horn)		
			4 (AK)		
			2 (SK)		
			2 (Lichen)		
			1 (CM)		
			1 (Nevi)		
			1 (Lip wart)		
			1 (Acrochordon)		
			1 (pyogenic granuloma)		
Total	88	9	60	157	1^a^

Abbreviations: AK, actinic keratosis; BCC, basal cell carcinoma; CM, cutaneous melanoma; DF, dermatofibroma; SCC, squamous cell carcinoma; SK, seborrheic keratosis.

^a^ Excluded from the cohort and statistics but included in this table only.

Out of the 772 correctly diagnosed BCCs and SCCs, 743 (96%) had a certain diagnosis with 655/743 (88%) BCCs and 88/743 (12%) SCCs, and 29 (4%) had a clinically uncertain diagnosis, out of which 20/29 (69%) were BCCs and 9/29 (31%) were SCCs. The difference between BCCs and SCCs is statistically significant χ^2^(1, *N* = 772) = 7.69, *p* = 0.005, OR = 3.35 [1.48, 7.59], confirmed with the Fischer exact test (*p* = 0.012). The clinical accuracy varied among physicians from 97% to 81%, and for BCCs and from 100% to 12% for SCCs (Table 2).

**TABLE 2:** Diagnosis accuracy and total number of cases for the dermatologists with the most cases and a grouping of those who had insufficient cases to be listed individually.

Physician	BCC accuracy	SCC accuracy (%)	Total BCC cases	Total SCC cases
1	92	72	38	11
2	96	58	356	82
3	97	66	148	24
4	94	100	68	8
5	95	100	21	6
6	81	12	16	8
7	95	55	21	9
Others	92	71	41	9
Total	95	62	709	157

Abbreviations: BCC, basal cell carcinoma; SCC, squamous cell carcinoma.

During the 2020 SARS‐CoV‐2 pandemic, the number of patients decreased by 41% from an average number of 169 for the previous 2 years to 99 patients in 2020. The mean thickness of excised tumors was significantly lower in 2021 and 2022 (M = 2.42, SD = 1.88, SEM = 0.11, *N* = 284) versus the 2 years prior to the pandemic (M = 2.04, SD = 1.49, SEM = 0.11, *N* = 182). The observed mean thickness difference was 0.38 mm, 95% CI [0.05, 0.70] (*p* = 0.023).

The most frequent location for both BCCs and SCCs with 551/866 (64%) was on the head (Table 3). Tumors located on the head were correctly diagnosed more often. Out of the 97 correctly diagnosed, 75 (74%) were located on the head, compared to 33/60 (55%) for misdiagnosed SCCs; the observed difference was statistically significant χ^2^(1, *N* = 157) = 7.59, *p* = 0.005, OR = 2.79 [1.39, 5.59], confirmed with the Fischer exact test (*p* = 0.006). Of the correctly diagnosed BCCs, 429/675 (64%) were localized on the face while 14/34 (42%) of the misdiagnosed BCCs were localized on the face, χ^2^(1, *N* = 709) = 5.99, *p* = 0.014, OR = 2.49 [1.24, 5.02], confirmed with the Fischer exact test (*p* = 0.016) (Table 4).

**TABLE 3:** Location of excised lesions.

		BCC	SCC
Location	Head	443	108
	Neck	68	—
	Torso	134	27
	Arms	30	8
	Legs	28	12
Unspecified		6	2

Abbreviations: BCC, basal cell carcinoma; SCC, squamous cell carcinoma.

**TABLE 4:** Analyzed factors with their clinical significance and statistical impact.

Analyzed factor	Significance	OR/mean, 95% CI	*p*
Advanced age	Predictive of SCC	6.96 years [4.89 years, 9.03 years]	< 0.001
Age 50 or under	Predictive of BCC	5.48 [1.76, 27.5]	0.002
Biopsied lesion	Predictive of SCC	2.13 [1.17, 3.75]	0.009
Multiple lesions	Predictive of BCC	2.16 [1.01, 4.78]	0.032
Correct diagnosis	Specific for BCC	12.28 [7.66, 19.68]	< 0.001
Clinical uncertainty	Predictive of SCC	3.35 [1.48, 7.59]	0.005
SCC on head	Better diagnosis	2.79 [1.39, 5.59]	0.005
BCC on head	Better diagnosis	2.49 [1.24, 5.02]	0.014
Three or more lesions	Superficial lesions	0.77 mm [0.32 mm, 1.22 mm]	< 0.001
Ulcerations	Predictive of BCC	2.14 [1.49, 3.08]	< 0.001
Thicker BCC	Predictive of BSC	1.01 mm [0.33, 1.69]	0.004

Abbreviations: BCC, basal cell carcinoma; OR, odds ratio and two standard deviation intervals; *p*, probability value; SCC, squamous cell carcinoma.

There were 137/678 (20%) patients with two or more lesions and 541/678 (80%) with a single excised lesion. 102/137 (74%) had two or more BCCs, 22/137 (16%) had a single SCC and one or more BCCs, and 13 (10%) had multiple SCCs.

The average tumor thickness was higher when a single lesion (M = 2.45, SD = 1.91, SEM = 0.08, *N* = 501) was excised versus when two or more lesions (M = 2.16, SD = 1.59, SEM = 0.13, *N* = 141) were excised, regardless of the nature of the second lesion, but without statistical significance (*p* = 0.092). The difference was larger when three or more lesions (M = 1.69, SD = 1.70, SEM = 0.19, *N* = 78) were excised. The difference was statistically significant, with a mean thickness difference of 0.77 mm; 95% CI [0.32, 1.22] was observed (*p* < 0.001).

The presence of more than three lesions was significantly specific for the presence of multiple BCCs than multiple SCCs or BBCs accompanied by an SCC χ^2^(1, *N* = 866) = 4.61, *p* = 0.032, OR = 2.16 [1.01, 4.78], confirmed with the Fischer exact test (*p* = 0.024). However, when single lesions versus two or more lesions were compared, the statistical significance was narrowly missed (*p* = 0.064).

There were 423 ulcerated lesions in our cohort. There was no observed difference between correctly 31/97 (31%) and falsely 22/60 (37%) diagnosed SCC (*p* = 0.665), or between correctly diagnosed BCCs 370/675 (55%) and falsely diagnosed BCCs 18/34 (53%) BCCs (*p* = 0.932). But ulcerations were more commonly found in BCCs than in SCCs χ^2^(1, *N* = 866) = 16.74, *p* < 0.001, OR = 2.14 [1.49, 3.08], confirmed with the Fischer exact test (*p* < 0.001).

Of the 157 SCCs, 50/157 (32%) were well differentiated, 25/157 (16%) were moderately differentiated, and 3/157 (2%) were poorly differentiated. The remaining 79/157 (50%) had no specification regarding differentiation. 26/709 (3.7%) of BCCs had squamous differentiation, and 22/26 (85%) were located in the head and neck area. They (M = 3.15, SD = 2.25, SEM = 0.45, *N* = 24) were characterized by a significantly increased tumor thickness of 1.01 mm, 95% CI [0.33, 1.69] compared to the rest of the BCCs (M = 2.14, SD = 1.63, SEM = 0.06, *N* = 616), (*p* = 0.004). Leucocytic infiltrate was the most common immune cell present in 445/866 (51%), and an immune infiltrate was observed in 506/866 (58%) of cases. A rich infiltrate was observed predominantly in cases where a single lesion was excised compared to the rest. No statistically significant relationship was found regarding the immune infiltrate.

## 4 Discussions

The mean age of our cohort was 69.88 years, with the mean age for BCC cases at 68.62 and 75.59 years for SCCs. In line with other studies such as Lee et al., showing NMSCs were more frequent in patients over 40, with a peak in 50 to 69 years old [15, 16]. Another study estimated the median and mean age at approximately 71 and 70.1 years for BCCs and 79 and 77.1 years for SCCs in the UK [17, 18]. According to Leman et al., 20% of all BCC cases occur in patients under 50 years old, significantly higher than the 10% rate observed in our cohort [19].

Although skin biopsies are not regularly performed in our department and the lesions are usually excised as a first intent, 13% of SCCs and 6.7% of BCCs were initially biopsied followed by an excision. The average thickness of biopsied SCCs was similar to that of all SCCs, but there was an observed difference in BCC cases. An article by Christensen et al. described a mean thickness of 1.53 and 1.67 mm for biopsied and excised BCCs. An explanation could be the larger number of cases in our cohort compared to the previous studies [20].

Not all lesions were precisely diagnosed before the surgery, but all lesions were excised based on a surgical protocol for a specific diagnosis, which represents the basis of our considerations for this article. The diagnostic accuracy for BCCs was significantly higher than that of SCCs and significantly more likely to be correctly diagnosed than SCC. Ryu et al. had similar observations to our study that SCCs have a higher tendency to be clinically misdiagnosed as BCCs than the opposite [21]. And also found that the diagnostic accuracy for BCC was higher than that of SCC. Ulceration, telangiectasia, and translucency were significant confusion factors. Another possible reason could be the fact that BCCs are more common than SCCs; therefore, physicians may have a tendency to diagnose an uncertain lesion as a BCC rather than an SCC.

A 41% decrease was observed in 2020 due to the SarsCoV‐19 pandemic. A retrospective study by Abbassi et al. reported a 158% increase of NMSC compared to the previous year. The increase was generated by an increased number of telemedicine consultations and reduced non‐urgent and non‐cancer elective surgeries [22]. In a meta‐analysis by Wall et al., a decrease in incidence in NMSC rates was observed, reported by all articles published in 2020–2021, compared to 2019. Only the incidence for BCC was diminished and not for SCC, mostly attributed to a delay in presentation, diagnosis, or treatment [23]. A peculiar observation observed in our study was the reduced thickness of tumors after the year 2020, which may be interpreted as a greater awareness of the need for medical care among patients or an increase in addressability. However, the trend would require more studies to confirm the trend.

The most frequent localization for both BCC and SCC was the head and neck, with 64% of cases. This being a common localization reported in the literature as well, the main risk factor for developing a BCC is sun exposure during childhood and/or adolescence [2, 24, 25, 26]. According to Neale et al., there is a modest correlation between sun exposure and the risk of developing a BCC, but SCCs are directly correlated with the quantity of sun exposure [27]. A retrospective study by Sgouros et al. reported that 23% have multiple BCCs, a higher number than our observations. They concluded that patients with multiple BCCs had a rich history of sun exposure and sunburns, and it is significantly lower than a 2019 review that summarized the role of genetic susceptibility and the greater prevalence of the superficial variant of BCC [28].

Ulcerations were more commonly found in correctly diagnosed BCC when examined clinically through dermoscopy and confirmed histopathologically. This finding is consistent with the study published by Hyung Ryu et al., together with other dermoscopic common traits of cBCC: blue‐gray nests and globules, arborizing vessels; the sensitivity of its diagnostic being greater than 95% [21]. The presence of ulceration in a cBCC lesion can also aid in its subtype differentiation; therefore, this trait is less consistent with a superficial BCC, as opposed to other clinical traits found when performing dermoscopy, which suggest a more likely diagnosis of sBCC (short‐line superficial telangiectasias, maple‐leaf like areas) [29].

BCCs with a squamous differentiation, or basosquamous carcinoma (BSC), are an aggressive type of basal cell carcinoma [30]. One of its similarities with basal cell carcinoma is the localization: a higher tendency to be localized in the head and neck area [31]. We observed a 3.7% incidence, significantly higher than historical reports. Schuller et al. reported an incidence of 1.2% in a cohort of 2565 NMSCs cases [32]. Similar to the 1.4% from another cohort from the University of Louisville between 1985 and 1988, with 31 lesions out of a total of 2075 cases [33]. With a higher incidence of 2.7% in the early 00′, and a 4.8% incidence in the early 10′ [34]. These results may suggest an increased incidence of BSC to over double the historical data, with significant clinical implications considering the risk of multiple tumors developing during the patient's lifespan [35].

## 5 Conclusion

Diagnosing NMSC can be a real challenge, especially when differentiating the two main representatives. With the increasing incidence of BSC, several questions need to be answered regarding the physiopathological mechanisms that lead to this variant of BCC. Several factors have been shown to influence the clinical diagnostic and probability of either one of the two NMSC. Patients under 50 rarely have an SCC, which in general is harder to diagnose and is determined as unknown lesions, and have a misdiagnosis more often. While BCCs are more likely to be associated with other skin lesions, younger age, ulcerations, and a higher risk towards developing further lesions. Lesions on the head are less likely to be misdiagnosed. SCC was misdiagnosed in about one third of cases, which should raise awareness of the clinical and dermoscopy pitfalls. Attending dermatologists should take into consideration the diagnosis of an SCC, especially when confusing factors are present such as a younger age of the patient, non‐facial lesions, or there is clinical uncertainty regarding the lesion. Ambiguous cases should be biopsied, especially in sensitive locations such as the facial area.

Overall, by integrating the clinical risk factors identified in clinical practice, dermatologists could enhance their preoperative diagnosis accuracy, reduce unnecessary procedures, better prioritize cases, and increase patient outcomes.

## Author Contributions

**Nicholas Florin Kormos:** conceptualization, investigation, writing – original draft, methodology, writing – review and editing, project administration, data curation, supervision. **Ioana Daria Paval:** conceptualization, data curation, methodology, writing – review and editing, writing – original draft, investigation, visualization. **Carina Maria Petrenciu:** conceptualization, investigation, writing – original draft, methodology, software, data curation, formal analysis. **Alin Stefan Vizitiu:** conceptualization, data curation, methodology, validation, writing – review and editing, writing – original draft, investigation. **Adrian Lucian Baican:** conceptualization, supervision, resources, investigation, writing – review and editing, methodology, validation, project administration.

## Ethics Statement

Approved by the Cluj Emergency County Hospital, approval no. 45182/26.11.2024.

## Consent

The authors have nothing to report.

## Conflicts of Interest

The authors declare no conflicts of interest.

## Data Availability

The data that support the findings of this study are available from the corresponding author upon reasonable request.
